# Supplementary material for: Are carbon emissions trading and green financial instruments synergistic? -Comprehensive quantitative research based on content analysis
Source: PLoS One. 2024 Mar 7;19(3):e0298601. doi: 10.1371/journal.pone.0298601 (PMC10919601; doi:10.1371/journal.pone.0298601)
Supplement: S1 Data — (ZIP) [file pone.0298601.s001.zip › Data/地级市Z_F工作报告.zip!/░▓╗╒/╕╖╤⌠╩╨/└┤╫╘╣1⁄2╓┌║┼í╛┬φ┐╦╩2╛▌═°í┐_2013.docx]

各位代表：

现在，我代表市人民政府，向大会报告政府工作，请予审议，并请市政协委员和其他列席人员提出意见。

一、2012年工作回顾

过去的一年，全市人民在中共阜阳市委的正确领导下，深入贯彻落实党的十七大、十八大及省第九次、市第四次党代会精神，紧紧围绕主题主线，牢牢把握“稳中求进、进中求快、快中求好”的工作总基调，全面加快工业化、城镇化和农业现代化进程，全市经济社会继续保持了平稳较快发展的良好态势。初步统计，全市生产总值980亿元，比上年增长12.1％；实现财政收入136.6亿元，增长18.7%；城镇居民人均可支配收入19120元，农民人均纯收入6100元，分别增长14.5%和19.5%。

一年来，主要做了以下几个方面的工作：

第一，围绕提升总量，大力促进经济较快发展。始终把加快发展作为第一要务，认真落实省政府30条稳增长意见，及时出台了27条政策措施。深入实施工业跨越工程，工业经济质量效益同步提升，规模以上工业增加值实现285亿元，增长17.5%。规模以上企业发展到689户，其中超亿元企业180户，工业经济效益综合指数达到295%。组织开展园区建设提升年活动，全市开发园区转型升级工作扎实推进，颍上工业园区扩区规划获得省政府批准。阜合现代产业园区起步区基本实现“七通一平”，福和家园安置小区和合肥工投、华泰食品等项目开工建设。切实强化农业基础地位，制定出台鼓励支持规模经营发展现代农业的暂行办法，扎实开展粮食生产“四大行动”，粮食总产达104.6亿斤、增长4.6%，实现“七连增、九连丰”，荣获全国粮食生产先进市称号。土地流转力度进一步加大，流转面积达185.2万亩、占耕地面积的22%。新增农民专业合作社803家、省级现代农业示范区3个。畜牧养殖业健康发展，创建省级以上标准化示范场36家，规模养殖比重达73%。着力打造商贸物流名城，临沂商城二期开工建设，香港财富广场、汇鑫瑞丽名城建成开业，天筑豪生酒店投入运营，颍泉区成为首批全省服务业综合改革示范区。认真落实各项消费促进政策，实现社会消费品零售总额450亿元、增长16.8％。金融保险业稳健运行，年末各类金融机构存款余额 1475亿元、贷款余额643亿元，分别增长18.5%和20.9%。

第二，围绕增强后劲，持续加强项目投资工作。始终把扩大投资作为经济增长的主要抓手，大力推进项目谋划和建设工作，全年固定资产投资520亿元，增长30%，“6611”工程项目完成投资365亿元、占年度计划的105%。口孜东矿、中能化工合成氨改造、贝克药业等重点项目建成投产，国机轴承、昊源化工航天炉等项目加快建设。105国道阜阳至太和段建成通车，102省道颍州西湖至临泉段、202省道阜阳至阜南段和阜新高速、阜六铁路、漯阜铁路快速推进。阜阳船闸建成通航，沙颍河航道整治和颍州港主体工程进展顺利。改造完成县乡公路220公里，加固改造农村公路危桥235座。阜阳机场航线增加到10条，全年旅客吞吐量超过20万人次。组织开展招商引资攻坚年和会展经济发展年活动，成功举办了第六届皖西北投资贸易洽谈会、第五届华药会和首届阜阳现代农业博览会等节会活动。全年引进市外项目1054个，实际到位资金506亿元，增长33%。实际利用外资1.6亿美元，增长14.7%。深入推进与央企、全国知名民企及皖粤对接合作，累计开工项目283个、完成投资441亿元。

第三，围绕扩容提质，努力加快城乡建设步伐。始终把推进新型城镇化进程作为加快发展的重要引擎，组织开展城市总体规划修编工作，编制完成了阜城11个片区控制性详规和综合交通、环卫设施等专项规划。进一步理顺城市规划建设管理体制，组建了“七大片区”建设指挥部，组团式推进阜城开发建设。人民路、河滨路改造完成，老阜颍路、七渔河路南段、双清路东段竣工通车，三清路、双清路西段、北三环下穿铁路立交工程加快推进。坚持群众利益优先、安置优先，碧水云天安置小区一期工程交付使用，阜阳经济技术开发区和谐新村、三角洲东岸和谐佳苑和界首路、南京路安置区加快建设。注重加强城市公共设施和绿化美化亮化工作，三角洲公园绿化工程基本完工，西城河公园、泉河风景带北岸绿化工程加快建设。全面启动国家级园林城市创建工作，颍上县荣获“国家园林县城”称号。改造完成40条小街巷，颍东污水处理厂建成使用，中水厂项目开工建设，天然气管网和加气站建设稳步推进。优先发展公交事业，新增40辆空调公交车、8辆双层公交车。组织开展市容环卫管理和城管执法督查考评工作，扎实推进砂石市场及交通秩序整治，市容市貌得到改善。加大城乡统筹力度，5县市建设步伐加快，扩权强镇试点扎实开展，美好乡村建设全面启动，“百村示范、千村整治”行动计划深入实施，农村危房改造及清洁工程进展顺利。

第四，围绕增添活力，着力加大改革开放力度。始终把改革创新作为推动发展的强大动力，出台扶持企业上市的政策措施，列入省上市后备企业达到8家。大力推进非公有制经济加快发展，全市非公企业和个体工商户分别达到2万家和13万户。认真实施营改增试点工作，全面推行市直预算单位公务卡制度改革，组织开展市直行政事业单位银行账户和经营性房产清理工作。进一步理顺市区财政管理体制，改革土地出让金征管办法。加大行政审批制度改革力度，市本级取消行政审批项目34项。深入推进医药卫生体制改革，县级公立医院综合改革全面展开。加快地方金融机构改革步伐，颍泉农村商业银行批准挂牌，颍州、界首农村信用联社改制方案获得批准。阜阳交通能源投资有限公司、城南新区投资有限公司组建运营。全市小额贷款公司、担保公司分别发展到24家和17家。组建了市招投标监督管理局和集中招投标交易平台，全面放开规划、建设、投融资市场。进一步扩大对外贸易，进出口总额首次突破10亿美元、增长70％，其中出口8.8亿美元、增长75%。

第五，围绕民计民生，全面发展社会各项事业。始终把改善民生作为经济发展的根本目的，启动实施城乡居民收入倍增计划，33项民生工程全面完成。高度重视就业再就业工作，大力促进高校毕业生、城镇就业困难人员和退役士兵就业，城镇登记失业率控制在4%以内。加大农民工就业创业工作力度，切实维护广大农民工的合法权益，春节期间举办了“接您回家”等系列活动。进一步加强城乡低保规范化管理，实行失业保险市级统筹，实现城乡居民养老保险制度全覆盖，5项社会保险超额完成全年目标任务。进一步提高最低工资及最低生活保障标准，完善社会救助保障与物价上涨挂钩联动机制，实行困难群众殡葬救助。推进农副产品平价商店建设，加强价格监管工作，全市居民消费价格指数控制在3%以内。深入推进科教兴阜战略，全年专利申请量超过2500件、授权量突破1000件。认真实施阜城中小学布点规划和学前教育三年行动计划，阜阳技师学院和阜阳职业技术学院、阜阳三中新校区加快建设，中小学校舍加固改造工程全面完成。进一步加强卫生服务体系建设，市人民医院、第六人民医院新区和第三人民医院住院大楼开工建设，新农合制度覆盖面进一步扩大。加强保障性住房建设，开工建设21445套，基本建成23624套，超额完成年度任务。深入实施文化惠民工程，乡镇综合文化站和农家书屋建设任务全面完成，广播电视、新闻出版、文学艺术进一步繁荣，我市荣获“中国书法城”称号。大力加强精神文明建设，全市54人入选“中国好人榜”。成功举办市首届体育大会，市体育中心综合馆主体工程竣工，我市籍运动员邓琳琳荣获第30届奥运会体操平衡木金牌。坚持计划生育基本国策，低生育水平持续稳定。组织实施新一轮扶贫开发攻坚规划。公布了第一批市级重点文物保护单位。妇女、儿童及老龄、老干部工作进一步加强。红十字、社会慈善及残疾人事业稳步发展。

第六，围绕和谐稳定，深入推进社会管理创新。始终把维护社会稳定作为第一责任，高度重视信访维稳工作，深入开展矛盾纠纷大排查，着力解决群众关注的热点难点问题，一批信访积案得到清理化解。深入开展安全生产年活动，扎实推进“打非治违”专项行动，加大校车安全排查整治力度，安全生产形势总体平稳。强化食品药品监管工作，努力提高食品卫生和药品安全保障水平。认真落实能源消费强度和总量双控制要求，单位生产总值能耗下降3%以上，主要污染物减排任务较好完成。大力发展循环经济，启动实施森林增长工程。加大闲置土地清理力度，认真处置阜城土地规划房产遗留问题。进一步完善应急管理体系，突发事件处置能力逐步增强。组织开展严打专项行动，社会治安形势持续好转。支持驻阜部队建设，武警、人防、民兵预备役和双拥优抚工作进一步加强。民族、宗教、统计、档案、地方志、地名、气象、地震、外事、侨务、港澳台、海关、出入境检验检疫等工作也都取得了新的成绩。

在加快经济社会发展的同时，我们注重加强政府自身建设，坚持市政府常务会议学法制度，深入推进“六五”普法工作。进一步完善政府目标管理考核体系，开展了规范性文件清理，出台了精简会议、文件和领导同志事务性活动的意见。深化政务公开工作，市政务服务中心入驻项目达417个。扎实推进机关效能建设，认真做好“12345”市长热线和网络问政工作。全面推行廉政风险防控制度，加强政府性投资审计，认真解决工程建设领域突出问题。开展公务用车、庆典研讨会论坛活动等专项治理，严格控制“三公”经费支出。强化重点领域、关键环节的行政监察、审计监督和政务督查。各级政府自觉接受人大法律监督和政协民主监督，认真办理人大代表议案、建议和政协委员提案，广泛听取各民主党派、工商联、无党派人士及各人民团体的建议和意见，鼓励支持人民群众、新闻媒体对政府工作进行监督，政风行风进一步好转。

在肯定成绩的同时，我们也清醒地认识到，我市经济社会发展中还存在一些不容忽视的困难和问题。一是发展不快、发展不足的问题依然突出，人均水平偏低，工业化、城镇化水平不高，农业基础较为薄弱。二是资金、土地、水资源等要素制约日益凸显，节能减排和生态环境建设压力明显加大，转变经济发展方式任务艰巨。三是社会事业发展相对滞后，公共服务和社会保障水平还不高，人民生活有待进一步改善，社会管理创新和维护和谐稳定工作面临新的挑战。四是政府效能建设和绩效管理水平还不高，形式主义、官僚主义和铺张浪费、消极腐败现象还不同程度地存在，发展环境有待进一步优化。对此，我们一定高度重视，采取更加有力的措施，认真加以解决。

各位代表，过去的一年，在面对诸多不利因素和挑战的情况下，我们能够取得这样的成绩，确实来之不易。这是省委、省政府和市委正确领导的结果，是市人大、市政协大力支持的结果，是全市广大干部群众齐心协力、团结奋斗的结果。在此，我代表市人民政府，向辛勤工作在全市各条战线上的广大干部群众，向给予政府工作大力支持与监督的人大代表、政协委员、各民主党派、工商联、无党派人士、各人民团体、新闻媒体和其他各界人士，向关心支持阜阳建设的离退休老干部、老职工，向为阜阳改革发展稳定作出贡献的解放军指战员、武警消防官兵、预备役部队、政法干警，向中央和省驻阜单位及所有关心支持阜阳建设与发展的同志们、朋友们，向在我市创业的境内外投资者、建设者，致以崇高的敬意和衷心的感谢！

二、2013年工作安排

今年是全面贯彻落实党的十八大精神的开局之年，是实施“十二五”规划承前启后的关键一年。我们既面临国家实施中原经济区建设、沿海地区产业加速转移、皖北振兴力度加大和大别山集中连片扶贫开发等众多机遇，也面临经济发展滞后、结构矛盾突出、区域竞争激烈等困难和挑战。但总体上机遇大于挑战，我们正处在大有作为的黄金发展期、工业化和城镇化加速推进期，到了必须加快发展也能够加快发展的关键阶段。只要全市上下坚定信心、抢抓机遇，开拓进取、扎实工作，就一定能够夺取科学发展、加速崛起的新胜利。

今年政府工作的指导思想是：认真学习贯彻党的十八大精神，以邓小平理论、“三个代表”重要思想、科学发展观为指导，全面落实市委四届五次全会和全市经济工作会议精神，立足打造“四个阜阳”和区域中心城市，紧紧围绕主题主线，切实把扩大总量作为重要目标，坚持增长速度与质量效益相统一，坚持改革开放和创新驱动，大力推进工业化、信息化、城镇化和农业现代化，努力扩大有效需求，着力优化产业结构，切实保障和改善民生，实现经济又好又快发展和社会和谐稳定，为全面建成小康社会奠定坚实基础。

全年经济社会发展的主要预期目标是：全市生产总值增长12%以上，财政收入增长15%以上，固定资产投资增长30%以上，社会消费品零售总额增长16%以上，进出口总额增长15%以上，城镇居民人均可支配收入增长13%以上，农民人均纯收入增长15%以上，城镇登记失业率控制在4%以内，完成省下达的节能减排年度任务。

实现上述目标任务，重点抓好以下八个方面的工作：

（一）切实加大工业强市推进力度，全面加快新型工业化进程。牢固树立工业立市理念，进一步加大主攻工业力度，确保规模以上工业增加值增长20%以上。一是着力培育壮大主导产业和骨干企业。切实加强工业经济运行调度，继续实行市级领导联系重点企业制度，突出抓好八大支柱产业。稳步扩大刘庄煤矿、谢桥煤矿、口孜东矿产能，支持昊源集团和晋煤中能化工加快发展，争取华润电厂二期及早获得国家核准，扎实做好刘庄煤矿坑口电厂及口孜西矿、罗园煤矿前期工作。加快各类食品工业园发展，推进金种子生态产业园项目建设，实施阜阳粮食产业园二期工程，支持面粉行业组建大型企业集团，促进阜阳卷烟厂、福润肉类、文王酒业等骨干企业做大做强。加快开乐专用车三期、阜阳轴承产业基地、中原电动车项目建设，巩固提升专用车、通用设备等重点行业。发挥华源医药集团市场优势，做大做强悦康、锦辉等医药企业，不断壮大生物医药产业。加快青岛即发、南源织造、豹子头服饰等企业发展，争取杉杉集团总部落户阜阳。以东盾、大亚、沪千木业和圣象地板等企业为龙头，大力发展林产品加工业。推广再生资源回收加工节能减排新技术，整合壮大华鑫集团、华翼集团、光武塑业集团等骨干企业实力。大力培育电子信息、新能源、新材料等新兴产业，推动产业转型升级。二是切实加大工业投资力度。突出抓好工业项目引进工作，建立市、县工业投资平台，努力扩大投资规模。大力开展银企对接活动，充分发挥融资平台和政府担保公司作用，支持贝克、华信药业等优势企业及早上市，鼓励企业采取票据、债券、信托等方式进行融资，力争全年工业投资增长20%以上。加大企业技术改造力度，计划实施技改项目317个，完成投资164亿元。加快国有企业改革步伐，推进华源纺织、金种子集团下属企业依法破产后续工作。全面运用信息技术改造传统产业，促进信息化、工业化深度融合，提高企业生产经营水平。三是加快推进园区转型升级。认真贯彻全省开发区转型发展工作交流会精神，加快扩区升级步伐，坚持集约节约用地，提高建设档次，增强综合承载能力。支持阜合现代产业园区完善基础设施，加快项目建设，优化体制机制，着力打造皖北地区“三化”协调发展的先导区、城乡统筹发展的现代化新城区。加快阜阳经济技术开发区建设步伐，重点发展优势产业和高新技术产业。全面推进县域开发园区和乡镇工业集聚区突出特色、完善规划，优化产业和空间布局,提升产业集中度和投资强度。四是大力发展民营经济。切实把民营经济摆上更加突出的战略位置，认真落实促进民营经济发展的政策措施，发挥民营经济发展专项资金杠杆作用，扩大中小企业投资担保规模，降低创业门槛，放宽市场准入，推动民营经济持续快速发展。

（二）切实加大中心城市建设力度，加速推进城乡发展一体化。围绕建设中原经济区东部门户和皖西北区域中心城市，按照加快新区建设、改造提升老城的思路，进一步加大投入，全面推进阜城大建设、大发展，走集约、智能、绿色、低碳的新型城镇化道路。一是突出科学规划引领。组织实施规划编制年活动，立足构建阜城特大城市、中小城市、特色乡镇、新农村四位一体的城乡发展体系，修编完成阜阳市城市总体规划，编制完成城南新区、阜合现代产业园区、颍州西湖控制性详规和教育、卫生等专项规划。高标准编制县城、中心镇和新农村发展规划。二是突出征迁安置工作。按照先安置后征迁的原则，坚持依法征迁、和谐征迁、廉洁征迁，在土地、资金、政策等方面优先保障安置房建设，解决好失地农民生产、生活问题，切实维护征迁群众的合法权益。加大各类保障性住房和安置房建设力度，推进和谐佳苑、南京路、碧水云天二期、职教园区、白衣桥和南城河安置小区建设。三是突出基础设施建设。全面加快阜城组团式发展步伐，推进城南新区、七渔河、职教园区路网建设，开工建设东三环路及颍河大桥，实施阜王路、涡阳路等道路延伸工程，促进北三环下穿铁路立交及早建成通车。加快建设三角洲公园书法广场，推进农民工广场、七渔河公园、刘锜公园和植物园建设，高标准实施淮河路、阜颍路等道路景观绿化工程。加快西城河景观带建设，推进七渔河、一道河、七里长沟和颍河、泉河城区段环境综合治理，实施“三清”河治理南延工程。加强小街巷治理工作，推进老城区危旧小区综合改造。进一步完善城市供水管网，开工建设南照淮河取水工程和颍州、泉北污水处理厂。四是突出城乡统筹发展。按照中等城市标准高起点建设县城，进一步拉大框架、完善功能、提升档次，加快与中心城市融合，不断壮大县域经济实力。扎实推进扩权强镇试点工作，今年再选择20个中心镇进行重点培育，不断提升建设品位和管理水平。加快户籍制度改革，有序推进农业转移人口市民化。五是突出管理水平提升。建立健全“两级政府、三级管理、四级网络”的城市管理责任体系，强化违法建设防控。加快市容环卫等基础设施建设，推进环卫管理体制改革，提高市容和环境卫生管理水平。加大“五城同创”力度，争创全国创建文明城市工作先进市、国家园林城市和第三届省文明城市。加强土地市场监管，深入开展闲置低效用地清理工作，加大土地收储和出让力度，进一步解决土地规划房产等遗留问题。加强交通秩序综合整治，大力发展城市公共交通事业，今年阜城再添置100辆空调公交车，开通阜阳至太和城际客运专线。

（三）切实加大现代农业发展力度，千方百计增加农民收入。认真落实中央农村工作会议精神，深化农村改革，创新农业生产经营体系，巩固百亿江淮粮仓地位，不断提升农村发展活力。一是着力提高农业组织化程度。加快转变农业发展方式，鼓励发展多种形式的新型农业合作组织，推进横向联合，实行分类指导，构建集约化、专业化、组织化、社会化相结合的新型经营体系。加大规模经营扶持力度，加快土地向种养大户和专业化组织集中，努力扩大土地流转规模。着力完善为农服务体系，建立健全农技人员与种养大户的利益联结机制，推进农资经营、农机作业、病虫防治、动物诊疗、产品销售等专业化服务，降低经营风险，提高生产经营效率。深入实施新型农民培训、创业富民和“凤还巢”工程，推进农民工创业园建设。继续开展春节期间“接您回家”活动，办好返乡农民工就业招聘洽谈会，促进农民工返乡就业创业。二是着力优化农业结构。继续实施粮食生产“四大行动”，依靠科技，主攻单产，提升品质，增加总产。大力发展高效经济作物，重点发展日光温室、工厂化大型温室等设施农业，加快发展城郊型农业、观光休闲农业，培育一村一品、一乡一业，创建一批特色农业示范区。统筹推进新一轮“菜篮子”工程建设，高标准改造一批城区农贸市场。加快发展生态健康养殖，深入开展标准化示范场创建工作，加强安全畜产品主体功能区建设，推进现代畜牧业发展。大力发展农业循环经济，促进农业资源综合利用。三是着力加快农业产业化步伐。深入实施农业产业化转型倍增计划，落实财政贴息和以奖代补政策，鼓励支持龙头企业扩大规模、扩建基地、延伸链条，提升档次和水平。重点扶持京九丝绸、海泉粮油、三宝线业、银海集团等龙头企业，支持颍东食品工业园争创国家级农业产业化示范区，推动农产品加工业向规模化、品牌化、集群化发展。大力开展现代农业示范区创建活动，争创国家级现代农业示范区1个、省级2个以上。四是着力改善农业生产条件。加强农业综合生产能力建设，强化科技服务，不断提升农业物质技术装备水平。推进新一轮治淮重点水利工程和中小河流治理工程，加快淮河流域重点平原洼地治理、行蓄洪区安全建设，完善防洪保安工程体系。大力实施土地整理复垦、农业综合开发项目，完成87万亩高标准基本农田建设任务，扩大节水灌溉和旱涝保收农田面积。认真落实农机补贴政策，积极推广新型农业机械。扎实开展新一轮扶贫开发攻坚，加快脱贫致富步伐。加强气象基础设施和人工影响天气作业能力建设。五是着力推进美好乡村建设。坚持规划引领、试点先行，整合各类涉农资源和资金，重点扶持176个中心村，治理改造1200个自然村，同步推进产业发展和社会管理，打造一批功能完备、设施齐全、环境优美的中心村。

（四）切实加大重点项目实施力度，着力增强经济发展后劲。今年初步安排“6611”工程项目597个，计划投资438亿元；其中省“861”行动计划项目225个，计划投资308亿元。一是扎实做好项目谋划争取工作。围绕国家政策和投资导向，立足阜阳产业基础优势，努力谋划编制一批牵动性强、综合效益高的重大项目，力争储备项目投资额超过4000亿元。加大项目争取力度，以市场换项目、以资源换项目，着力引进一批带动能力强、投资规模大、有利于产业转型升级的战略项目。二是扎实做好重点项目实施工作。进一步完善项目推进工作机制，加快重点项目建设步伐，开工建设阜城外环快速通道和市县快速通道阜阳至颍上段、太和至界首段、太和三桥连接线至三角元段，实施阜阳至杨楼孜道路升级改造、阜新高速临泉连接线、杨桥船闸扩建项目，加快建设漯阜铁路、阜六铁路、阜淮三线，推进阜阳机场通航能力改造提升、沙颍河航道整治和闻集500千伏输变电工程，完成阜新高速、102省道颍州西湖至临泉段、202省道阜阳至阜南段和颍州港区一期工程，力争开工建设阜阳船闸、颍上船闸复线。加快阜阳至淮滨高速公路、商阜杭高速铁路和漯阜铁路临泉支线等项目前期工作，争取早日开工。加固改造农村公路危桥243座，改造完成县乡公路330公里。三是扎实做好招商引资工作。坚持“大抓招商、抓招大商”，采取领导同志定期外出招商、小分队驻外定点招商、聘请专业人员招商、行业协会招商等方式，加大奖励激励力度，强化跟踪服务，切实提升招商引资质量和水平。认真组织参加徽商大会、中博会、西洽会等招商活动，继续办好皖西北贸洽会、华药会、现代农业博览会，支持各地举办各具特色的节会活动。四是扎实做好对接合作工作。认真落实与央企、全国知名民企及皖粤经贸合作签约项目，瞄准央企和知名民营企业的战略投资意图和合作需求，力争引进一批产业层次高、带动能力强的重大项目。深化与合肥结对合作，不断巩固扩大成果。加强与中原经济区兄弟市的互利合作。

（五）切实加大商贸市场培育力度，大力发展现代服务业。围绕构建商贸物流名城，进一步做大市场、做活商圈、做强物流，加快发展第三产业。一是加快商贸物流市场建设。编制商业网点建设中长期规划，完善提升人民路和西二环路核心商圈辐射带动功能。加快临沂商城二期、阜阳汽贸物流园、阜阳农机大市场等专业化市场建设。高标准改造皖西北商贸城，建设阜阳四季青国际服装城。发展壮大仓储物流业，鼓励发展第三方物流和电子商务，扎实推进华源医药、阜阳烟草、阜阳商厦、中储粮农产品等物流园建设。二是积极扩大消费需求。深入实施“双百市场工程”、“万村千乡市场工程”、“新网工程”，做好节能惠民工程等政策接续工作，积极培育家政养老、休闲健身、节能环保产品等消费热点，推动发展网络购物等新型消费业态。抓住营改增试点机遇，加快发展现代物流、科技、信息、金融等生产性服务业，全面提升商贸、餐饮、房地产等生活性服务业，积极培育服务外包、文化创意、会展经济等新兴服务业。支持颍州西湖、颍上八里河、阜阳生态园等风景区完善基础设施，加大宣传推介力度，促进旅游业快速发展。三是努力提升对外贸易水平。认真落实促进对外贸易的各项政策措施，积极推进口岸建设，加快发展海铁联运，培育壮大人发、柳编、洁具、渔具、香料、调味品等出口基地建设，着力扩大纺织、服装、特色农产品等优势产品出口，进一步提高经济外向度。

（六）切实加大财政金融工作力度，不断提升经济保障能力。认真落实积极的财政政策，加快推进财政金融改革与创新，更好地支持和服务经济社会发展。一是持续增强财政保障能力。大力培植财源，完善税源动态监控机制，提高税收征管水平。认真落实结构性减税政策，扩大营改增试点范围。进一步理顺市区财政、土地出让金管理和养老统筹体制，形成有利于调动各方积极性的工作机制。优化财政支出结构，更多地向民生倾斜、向基层倾斜、向弱势群体倾斜。深入推进预算管理制度改革，加强财政资金监管和政府性债务管理，提高资金使用效益。二是切实提升金融服务水平。进一步加强银企合作，引导金融机构推进金融产品和服务方式创新，加大对战略性新兴产业、重大基础设施的信贷支持力度，扩大对“三农”、中小微企业等贷款规模，力争全年新增贷款130亿元以上。加强担保体系建设，规范发展融资性担保公司。支持有条件的企业发行企业债、短期融资债和中期票据，探索发行中小企业集合债，开展土地使用权抵押贷款试点。三是加快推进地方金融体系建设。大力支持各类银行保险证券机构加强服务网点建设，争取更多的股份制商业银行在阜设立分支机构。加快颍淮、界首农村商业银行组建工作，力争临泉、太和、阜南、颍上农村商业银行获批筹建。大力发展村镇银行，规范小额贷款公司经营行为。加强金融生态和社会信用体系建设，进一步优化金融生态环境。

（七）切实加大社会事业统筹力度，倾力改善民计民生。加快发展社会各项事业，大力推进公共服务均等化，着力提升人民群众的生活质量和幸福指数。一是深入实施科教兴阜战略。加大政府科技投入，加快构建技术创新体系，促进科技成果转化，发挥科技创新对产业升级的核心作用。优先发展教育事业，建立教育融资平台，实施阜阳城区基础教育三年提升计划，加快义务教育薄弱学校标准化建设，逐步解决城区学校“择校热”和“大班额”等问题。全面完成学前教育三年行动计划，加大阜阳幼儿师范高等专科学校创建力度。着力提高普通高中办学能力，确保阜阳三中新校区建成投入使用。扎实推进职教大市建设，整合职业教育资源，实施阜阳职教园区建设规划，加快阜阳技师学院和阜阳职业技术学院新校区建设步伐。关心支持特殊教育，加快发展成人教育，鼓励发展民办教育。继续做好家庭经济困难学生资助和留守儿童关爱工作。二是加强民生改善和社会保障。按照省统一部署，继续实施33项民生工程，着力提高民生工程的使用效益，使发展成果更多、更公平惠及全市人民。认真实施更加积极的就业政策，突出抓好高校毕业生、城镇就业困难人员、农村劳动力和退役士兵就业工作。实施城乡低保标准和补差水平双倍增规划，保障城乡困难居民基本生活。规范养老、医疗、失业保险统筹，完善养老保险转移接续和失地农民参保办法，进一步提高城乡居民养老保险水平。按照做大做强公立医院、培植培育民办医疗、深化完善基层医改的要求，制定实施医疗卫生事业三年发展计划，力争2015年全市人均拥有床位数提高1个千分点以上。深化医药卫生体制改革，全面推进县级公立医院综合改革，加快医疗卫生服务体系建设，着力提高医疗服务质量，切实解决看病难、看病贵和看病不在当地的问题。认真开展价格调控和监管，保持价格总水平基本稳定。三是大力发展文化体育事业。扎实推进社会主义核心价值体系建设，深入开展群众性精神文明创建活动。加大重大文化工程和公共场馆建设力度，规划建设市规划馆、博物馆、图书馆、科技馆、文化馆、书法艺术馆、大剧院和烈士纪念馆，建成市体育中心综合馆和少年宫二期工程。大力开展文艺精品创作和群众文化活动，全面实行公共文化设施免费开放。加强非物质文化遗产保护和开发利用工作。广泛开展全民健身运动，认真筹办市第12届运动会，备战省第13届运动会。四是扎实推进生态文明建设。加快实施森林增长工程，高标准建设阜太路绿色长廊，完成成片造林12.24万亩。加强农村环境连片整治，开展国家级、省级生态乡镇、生态村创建工作。实行最严格的水资源管理制度，促进水资源可持续利用。强化节能减排目标责任制，加强淮河流域水污染治理，抓好涉铅等重金属企业整治工作，大力推进机动车环保标志管理，加快建设阜阳华润电厂二号机组脱硝工程。严格实行能源消费强度和总量双控制，实施节能改造、合同能源管理等重点工程，坚决淘汰落后产能。大力发展绿色经济、低碳经济和循环经济，推进生态阜阳建设。五是做好人口和计划生育工作。认真落实计划生育奖励优惠政策，全面开展免费孕前优生健康检查，着力提高出生人口素质，降低出生人口性别比，促进人口长期均衡发展。同时，认真实施新一轮妇女儿童发展规划纲要。落实殡葬惠民政策，推进殡葬改革。加强残疾人社会保障和服务体系建设。

(八)切实加大社会管理创新力度，全力维护和谐稳定大局。高度重视社会稳定工作，不断提高社会管理水平，巩固和谐稳定的良好局面。一是加强社会管理工作。认真落实领导干部带案下访、开门接访制度，努力化解信访积案，切实维护群众合法权益。进一步完善人民调解、行政调解、司法调解联动工作体系，建立健全社会稳定风险评估机制，广泛开展矛盾纠纷大排查大调处，努力从源头上化解社会矛盾，有效预防和妥善处置群体性事件。二是抓好应急管理和安全生产。进一步健全应急管理体制机制，强化应急管理基础建设，不断提高信息预警和应急处置能力。全面落实安全生产责任制，深入开展“打非治违”和超载超限专项治理行动，加强校车安全隐患排查，加大对道路交通、建筑施工、危险化学品、人员集聚场所等重点领域隐患排查治理工作，严防重特大事故的发生。强化食品药品监管，建立健全农村食品药品监管网络体系，建设市食品药品检验检测中心，努力保障食品药品安全。三是深化平安创建活动。完善社会治安防控体系，加强社会治安综合治理，坚决打击各类违法犯罪。加强流动人口的服务和管理，全面实行居住证制度。加快完善社区综合管理体制，强化社区警务、群防群治和农村基层治保调解组织建设，维护社会和谐稳定。进一步加强统计基层基础工作，认真开展第三次全国经济普查。深入实施“六五”普法。大力支持驻阜部队现代化建设，创新推动国防动员、后备力量建设和人民防空工作。高度重视国家安全、保密和双拥工作。支持工会、共青团、妇联、关工委、老龄委工作。切实做好海关、出入境检验检疫、档案、地震、气象、地名等工作。积极推进民族、宗教、外事、侨务、港澳台、地方志、红十字和社会慈善等各项工作。

三、加强政府自身建设

全面建成小康社会的宏伟目标，对政府工作提出了新的更高的要求。各级政府及其工作人员务必按照为民、务实、清廉的要求，切实加强自身建设，着力提升推进科学发展的能力和水平。

（一）注重加强学习，进一步推进改革创新。牢固树立重视学习、坚持学习、终身学习的理念，切实养成爱读书、读好书的习惯。认真学习党的十八大精神和上级方针政策，学习履行职责必备的各类知识，努力在学习中开阔眼界、把握规律、培养战略思维，切实提升研判形势、把握大势、驾驭全局的能力，真正成为领导经济工作、推进“四化”进程的行家里手。进一步解放思想、开拓创新，坚决破除影响加快发展的思想障碍，坚决摒弃束缚发展的条条框框，坚决革除阻碍发展的陈规陋习，以更广的眼界谋划发展，以更大的气魄推进发展，以更实的举措加快发展。大力弘扬“敢于啃硬骨头、敢于涉险滩”的精神，进一步加大改革开放力度，全面加快行政体制、金融服务、城市建设等重点领域改革进程，努力形成有利于加快发展的体制机制，凝聚起加速崛起的强大合力，勇当皖北崛起的排头兵。

（二）强化法治意识，进一步推进依法行政。各级政府要严格依照法定权限和程序行使权力、履行职责，切实做到按制度办事、按程序办事、按规矩办事。进一步规范行政执法行为，推进公正执法、文明执法。健全重大决策专家论证、集体审定、风险评估、社会公示和问责纠错制度，推进政府决策科学化、民主化、规范化。进一步深化政务公开，加强政务服务体系和电子政务建设，完善政府公共信息平台，积极推进网上审批、网络问政。各级政府及其工作人员要始终坚持党的领导，自觉维护党委权威，主动接受人大法律监督和政协民主监督，严格执行人大及其常委会的决议、决定，认真办理人大代表议案、建议和政协委员提案，广泛听取各民主党派、工商联、无党派人士和各人民团体的意见，鼓励支持人民群众、新闻媒体对政府工作进行监督。

（三）坚持执政为民，进一步推进作风转变。牢固树立以人为本、民生优先的理念，切实做到“服务才是管理，为民才能执政”。坚持问政于民、问需于民、问计于民，扎实推进基层走访活动制度化、常态化，更好地为人民服务、让人民满意。始终把群众安危冷暖放在心上，更加关注弱势群体，更加关心群众疾苦，着力解决事关人民群众切身利益的热点难点问题。牢记“实干兴邦、空谈误国”，认真落实中央改进工作作风“八项规定”和省委30条要求，进一步精简会议和文件，切实改进会风，减少事务性应酬。各级政府领导干部要带头深入基层，深入群众，真抓实干，各项工作都要有目标、有措施、有时间节点、有结果，只划“句号”、不划“逗号”，做到讲实话、干实事，敢作为、勇担当，言必信、行必果。切实加强行政效能建设，加大经济环境治理力度，努力营造良好的发展环境。

（四）大力从严治政，进一步推进廉政建设。严格落实党风廉政建设责任制，加强反腐倡廉教育，全面推进惩治和预防腐败体系建设，做到干部清正、政府清廉、政治清明。深入开展廉政风险防控工作，深化预算执行和领导干部经济责任审计工作，强化对权力运行的监督制约，建设电子监察系统，着力从源头上防治腐败。切实加大招投标监管力度，建立完善阳光、透明、高效、规范的交易平台。牢固树立艰苦奋斗、勤俭节约的思想，认真执行厉行节约各项规定，压缩“三公”经费支出，巩固公车治理成果，规范行政事业单位银行账户和经营性房产管理。各级领导干部要时刻牢记“两个务必”，带头执行廉洁自律的各项规定，要求别人做到的自己首先做到，要求别人不做的自己坚决不做，勤勉尽责、秉公用权，当廉洁奉公的表率，做人民满意的公仆。

各位代表，推进阜阳加速发展，实现奋力崛起，是时代赋予我们的光荣使命。让我们紧密团结在以习近平同志为核心的党中央周围，高举中国特色社会主义伟大旗帜，在中共阜阳市委的坚强领导下，进一步坚定信心，振奋精神，团结拼搏，扎实苦干，为全面建成小康社会而努力奋斗！
